# Using the K-Nearest Neighbor Algorithm for the Classification of Lymph Node Metastasis in Gastric Cancer

**DOI:** 10.1155/2012/876545

**Published:** 2012-10-24

**Authors:** Chao Li, Shuheng Zhang, Huan Zhang, Lifang Pang, Kinman Lam, Chun Hui, Su Zhang

**Affiliations:** ^1^School of Biomedical Engineering, Shanghai Jiao Tong University, Shanghai 200030, China; ^2^Department of Radiology, Ruijin Hospital, Shanghai Jiao Tong University School of Medicine, Shanghai 200025, China; ^3^Centre for Signal Processing, Department of Electronic and Information Engineering, The Hong Kong Polytechnic University, Hong Kong

## Abstract

Accurate tumor, node, and metastasis (TNM) staging, especially N staging in gastric cancer or the metastasis on lymph node diagnosis, is a popular issue in clinical medical image analysis in which gemstone spectral imaging (GSI) can provide more information to doctors than conventional computed tomography (CT) does. In this paper, we apply machine learning methods on the GSI analysis of lymph node metastasis in gastric cancer. First, we use some feature selection or metric learning methods to reduce data dimension and feature space. We then employ the K-nearest neighbor classifier to distinguish lymph node metastasis from nonlymph node metastasis. The experiment involved 38 lymph node samples in gastric cancer, showing an overall accuracy of 96.33%. Compared with that of traditional diagnostic methods, such as helical CT (sensitivity 75.2% and specificity 41.8%) and multidetector computed tomography (82.09%), the diagnostic accuracy of lymph node metastasis is high. GSI-CT can then be the optimal choice for the preoperative diagnosis of patients with gastric cancer in the N staging.

## 1. Introduction

According to the global cancer statistics in 2011, an estimated 989,600 new stomach cancer cases and 738,000 deaths occurred in 2008, which account for 8% of the total cases and 10% of the total deaths. Over 70% of the new cases and deaths were recorded in developing countries [1, 2]. The most commonly used staging system is the American Joint Committee on Cancer Tumor, Node, and Metastasis (TNM) [3–5]. The two most important factors that influence survival among patients with resectable gastric cancer are the depth of cancer invasion from the gastric wall and the number of lymph nodes present. In areas not screened for gastric cancer, late diagnosis reveals a high frequency of nodal involvement. Even in early gastric cancer, the incidence of lymph node metastasis exceeds 10%. The overall incidence was reported to be 14.1% and 4.8% to 23.6% depending on cancer depth [6]. The lymph node status must be pre-operatively evaluated for proper treatment. However, the various modalities could not obtain sufficient results. The lymph node status is one of the most important prognostic indicators of poor survival [7, 8].

Preoperative examinations, endoscopy, and barium meal examinations are routinely used to evaluate cancerous lesions in the stomach. Abdominal ultrasound, computed tomography (CT) examination, and magnetic resonance imaging (MRI) are commonly used to examine the presence of invasion to other organs and metastatic lesions. However, their diagnostic accuracy is limited. Endoscopic ultrasound has been the most reliable nonsurgical method in the evaluation of the primary tumor with 65% to 77% accuracy of N staging due to the limited penetration ability of the ultrasound for lymph node distant metastasis. In spite of the higher image quality and dynamic contrast-enhanced imaging, MRI only has an N staging accuracy of 65% to 70%. The multidetector row computed tomography (MDCT) [9] scanner enables for thinner collimation and faster scanning, which markedly improves imaging resolution and enable rapid handling of image reconstruction. Moreover, intravenous bolus administration of contrast material permits precise evaluation of carcinoma enhancement, and the water-filling method enables negative contrast to enhance the gastric wall. Thus, MDCT has a higher N staging accuracy of up to 82% and has become a main examination method for preoperative staging of gastric cancer [10]. Fukuya et al. [11] showed in their study for lymph nodes of at least 5 mm that sensitivity for detecting metastasis positive nodes was 75.2% and specificity for detecting metastasis negative nodes was 41.8%. A large-scale Chinese study [10] conducted by Ruijin Hospital showed that the overall diagnostic sensitivity, specificity, and accuracy of MDCT for determining lymph node metastasis was 86.26%, 76.17%, and 82.09%, respectively. However, with clinically valuable scanning protocols of the spectral CT imaging technology, we can obtain more information with gemstone spectral imaging (GSI) than with any conventional CT (e.g., MDCT). 

In conventional CT imaging, we measure the attenuation of the X-ray beam through an object. We commonly define the X-ray beam quality in terms of its kilo voltage peak (kVp) that denotes the maximum photon energy, as the X-ray beam comprises a mixture of X-ray photon energies. GSI [12] with spectral CT, and conventional attenuation data may be transformed into effective material densities, that enhance the tissue characterization capabilities of CT. Furthermore, through the monochromatic representation of the spectral CT, the beam-hardening artifacts can be substantially reduced, which is a step toward quantitative imaging with more consistent image measurements for examinations, patients, and scanners.

In this paper, we intend to use the machine learning method to handle the large amount information provided by GSI and to improve the accuracy for the determination of lymph node metastasis in gastric cancer. 

The paper is arranged as follows, Section 2 describes the details of the methods used in this paper, Section 3 presents the experimental framework and the results, and Section 4 concludes the present study and discusses potential future research. 

## 2. Methodology

**Figure 1:**
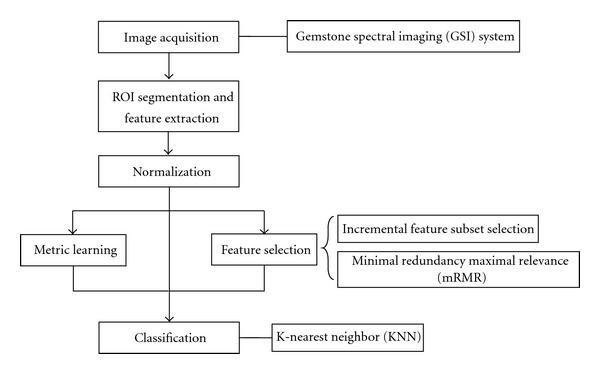
Flow chart classification on lymph node metastasis in gastric cancer.

Figure 1 shows a flow chart illustrating the whole framework of the classification on lymph node metastasis in gastric cancer. 

### 2.1. Pre-Processing

GSI-CT examination was performed among patients using the GE Discovery CT750 HD (GE-Healthcare) scanner [13]. Each patient received an intramuscular administration of 20 mg of anisodamine to decrease peristaltic bowel movement and drank 1,000 to 1,200 mL tap water for gastric filling 5 to 10 min before the scan. Patients were in a supine position. After obtaining the localizer CT radiographs (e.g., anterior-posterior and/or lateral), we captured the unenhanced scan of the upper abdomen and then employed the enhanced GSI scan in two phases. An 80 mL to 100 mL bolus of nonionic iodine contrast agent was administered to the ante-cubital vein at a flow rate of 2 mL/sec to 3 mL/sec through a 20-gauge needle using an automatic injector. CT acquisitions were performed in the arterial phase (start delay of 40 s) and in the portal venous phase (start delay of 70 sec). The arterial phase scans the whole stomach and the portal venous phase examines from the top of the stomach diaphragm to the abdominal aortic bifurcation plane. The GSI-CT scanning parameters are as follows: scan mode of spectral imaging with fast tube-voltage switching between 80 kVp and 140 kVp, the currents of 220 mA to 640 mA, slice thickness of 5 mm, rotation speed of 1.6 s to 0.8 s, and pitch ratio of 0.984 : 1.

### 2.2. Feature Extraction

**Figure 2:**
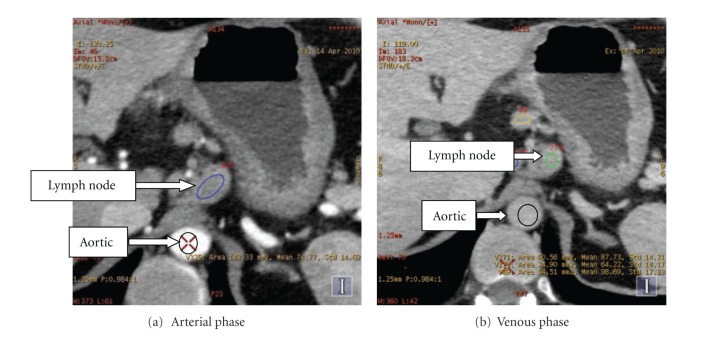
Gastric lymph node at 70 keV energy.

Lymph node regions of interest (ROIs) were delineated by experienced doctors. Not all the lymph nodes could be captured in the images because of the node size or location.  Figure 2 shows lymph node and aortic in the arterial phase and venous phase under 70 keV monochromatic energy. The lymph node on Figure 2(b) is difficult to find for its small size. The monochromatic values (Hu) and the mean of material basis pairs (*μ*g/cm^3^) were calculated. The features used in this paper are monochromatic CT values (40 keV to 140 keV) and material basis pairs (Calcium-Iodine, Calcium-Water, Iodine-Calcium, Iodine-Water, Water-Calcium, Water-Iodine, Effective-Z).

During the image acquisition process, variations on the injection speed, dose of the contrast agents and their circulation inside the body of patients can cause differences in the CT numerical values. To eliminate discrepancies, the arterial CT value of the same slice was recorded at mean time, and then normalization work was conducted by using the following formula:

(1) $$\begin{array}{c} \text{Norm}=\frac{\text{ROI}\;\;\text{mean}\;\;\text{CT}\;\;\text{value}}{\text{Aortic}\;\;\text{mean}\;\;\text{CT}\;\;\text{value}}. \end{array}$$

### 2.3. Feature Selection

#### 2.3.1. mRMR Algorithm

Minimal redundancy maximal relevance (mRMR) is a feature-selection scheme proposed by [14] mRMR that uses the information theory as a standard with better generalization and efficiency and accuracy for feature selection. Each feature can be ranked based on its relevance to the target variable, and the ranking process considers the redundancy of these features. An effective feature is defined as one that has the best trade-off between minimum redundancy within the features and maximum relevance to the target variable [15]. Mutual information (MI), which measures the mutual dependence of two variables, is used to quantify both relevance and redundancy in this method [16]. The two most used mRMR criteria are mutual information difference (MID) and mutual information quotient (MIQ),

(2) $$\begin{array}{c} \text{MID}:\underset{i\in \Omega_{s}}{\max}\left[H\left(i,c\right)-\frac{1}{\left|S\right|}\sum_{j\in S}H\left(i,j\right)\right],\\ \text{MIQ}:\underset{i\in \Omega_{s}}{\max}\left\{\frac{H\left(i,c\right)}{\left[\left(1/\left|S\right|\right)\sum_{j\in S}H\left(i,j\right)\right]}\right\}, \end{array}$$

where *H*(*i*, *c*) is the MI between feature *i* and classification *c*, *H*(*i*, *j*) is MI between features *i* and *j*, *S* is the current feature set, and |*S*| is the length of the feature set.

#### 2.3.2. SFS Algorithm

Sequential forward selection (SFS) is a traditional heuristic feature selection algorithm [17, 18]. SFS starts with an empty feature subset *S*_*i*_. In each iteration only one feature is added to the feature subset. To determine which feature to add, the algorithm tentatively adds an unselected feature to the candidate feature subset and tests the accuracy of the classifier built on the tentative feature subset. The feature that exhibits the highest accuracy is finally added to the feature subset. The process stops after an iteration in which no features can be added, resulting in an improvement in accuracy.

### 2.4. Metric Learning Algorithm

Learning good distance metrics in feature space is crucial to many machine learning works (e.g., classification). A lot of existing works has shown that properly designed distance metrics can greatly improve the KNN classification accuracy compared to the standard Euclidean distance. Depending on the feasibility of the training samples, distance metric learning algorithms can be divided into two categories: supervised distance metric learning and unsupervised distance metric learning.  Table 1 shows the several distance metric learning algorithms. Among them, principal component analysis (PCA) is the most commonly used algorithm for the problem of dimensionality reduction of large datasets like in the application of face recognition [19], image retrieval [20].

### 2.5. Classification

The K-nearest neighbor (KNN) [21, 26] algorithm is among the simplest of all machine algorithms. In this algorithm, an object is classified by a majority vote of its neighbors. The object is consequently assigned to the class that is most common among its KNN, where *K* is a positive integer that is typically small. If *K* = 1, then the object is simply assigned to the class of its nearest neighbor.

The KNN algorithm is first implemented by introducing some notations *S* = (*x*_*i*_, *y*_*i*_), *i* = 1,2,…*N* is considered the training set, where *x*_*i*_ is the *d*-dimensional feature vector, and *y*_*i*_ ∈ {+1, −1} is associated with the observed class labels. For simplicity, we consider a binary classification. We generally suppose that all training data are iid samples of random variables (*X*, *Y*) with unknown distribution. 

With previously labeled samples as the training set *S*, the KNN algorithm constructs a local subregion *R*(*x*)⊆*ℜ* ^*d*^ of the input space, which is situated at the estimation point *x*. The predicting region *R*(*x*) contains the closest training points to *x*, which is written as follows:

(3) $$\begin{array}{c} R\left(x\right)=\left\{\hat{x}\;|\;D\left(x,\hat{x}\right)\leq d_{\left(k\right)}\right\}, \end{array}$$

where *d*_(*k*)_ is the *k*th order statistic of ${\left\{D(x,\hat{x})\right\}}_{1}^{N}$, and $D(x,\hat{x})$ is the distance metric. *k*[*y*] denotes the number of samples in region *R*(*x*), which is labeled *y*. The KNN algorithm is statistically designed for the estimation of posterior probability *p*(*y* | *x*) of the observation point *x*:

(4) $$\begin{array}{c} p\left(y\;|\;x\right)=\frac{p\left(x\;|\;y\right)p\left(y\right)}{p\left(x\right)}≅\frac{k\left[y\right]}{k}. \end{array}$$

For a given observation *x*, the decision *g*(*x*) is formulated by evaluating the values of *k*[*y*] and selecting the class that has the highest *k*[*y*] value

(5) $$\begin{array}{c} g\left(x\right)=\{\begin{array}{cc} 1, & k\left[y=1\right]\geq k\left[y=-1\right],\\ -1, & k\left[y=-1\right]\geq k\left[y=1\right]. \end{array} \end{array}$$

Thus, the decision that maximizes the associated posterior probability is employed in the KNN algorithm. For a binary classification problem in which *y*_*i*_ ∈ {+1, −1}, the KNN algorithm produces the following decision rule:

(6) $$\begin{array}{c} g\left(x\right)=\mathrm{sgn}\left(\text{av}{\text{e}}_{x_{i}\in R(x)}y_{i}\right). \end{array}$$

## 3. Experimental Results and Discussion 

### 3.1. Experiments

The image data used in our work were acquired from GE Healthcare equipment in Ruijin Hospital on April 2010. We collected got 38 gastric lymph node datasets. Among the datasets were 27 lymph node metastasis (positive) and 11 nonlymph node metastasis (negative). All the lymph node data were pathology results obtained after lymph node dissection (lymphadenectomy) in patients.

#### 3.1.1. Univariate Analysis

In this study, we conduct univariate analysis by exploring variables (features) one by one. We analyze each feature by calculating its relevance to lymph node metastasis. Here, we use the following measurements:

(i) *Two-Tailed t-test*: The two-tailed test is a statistical test used in inference, in which a given statistical hypothesis, H0 (the null hypothesis), is rejected when the value of the test statistic is either sufficiently small or sufficiently large.
(ii) *Point Biserial Correlation Coefficient* (*r*_pb_):
  (7) $$\begin{array}{c} r_{\text{pb}}=\frac{\text{Av}{\text{g}}_{p}-\text{Av}{\text{g}}_{q}}{\text{St}{\text{d}}_{y}}\sqrt{pq}. \end{array}$$
  In regard to the *p*, *q* notation formula Avg_*p*_ is the mean for nondichotomous values in connection with the variable coded 1, and Avg_*q*_ is the mean for the non-dichotomous values for the same variable-coded 0. Std_*y*_ is the standard deviation for all non-dichotomous entries, and *p* and *q* are the proportions of the dichotomous variable-coded 1 and 0, respectively.
(iii) * Information Gain (IG)*: IG is calculated by the entropy of the feature *X*, *H*(*X*) minus the conditional entropy of *Y* given *X*, *H*(*X* | *Y*)
  (8) $$\begin{array}{c} \text{IG}\left(X\;|\;Y\right)=H\left(X\right)-H\left(X\;|\;Y\right). \end{array}$$
(iv) *Area Under Curve (AUC).*
(v) *Symmetrical Uncertainty (SU)*: SU is the normalization of IG within [0, 1], where the higher value of SU shows a higher relevance for feature *X* and class *Y* (as a measure of correlation between the features and the concept target)
  (9) $$\begin{array}{c} \text{SU}\left(X,Y\right)=2\left[\frac{IG\left(X\;|\;Y\right)}{H\left(X\right)+H\left(Y\right)}\right]. \end{array}$$

The experimental results of the univariate analysis are shown in Tables 2 and 3. Based on the table, the Iodine-Water, Iodine-Calcium, Calcium-Iodine, and Effective-Z features show high relevance to lymph node metastasis. Among these features, high relevance to lymph node metastasis was clinically confirmed for Iodine-Water and Effective-Z features. Both Iodine-Water and Iodine-Calcium features reflect the concentration of the iodinated contrast media uptake by the surrounding tissue, and thus they are related to lymph node metastasis. The Calcium-Iodine feature indicates tissue calcification, which rarely exists in lymph nodes. However, experimental results show that the Calcium-Iodine feature is highly related to lymph node metastasis, which must be further verified by clinical results.

**Figure 3:**
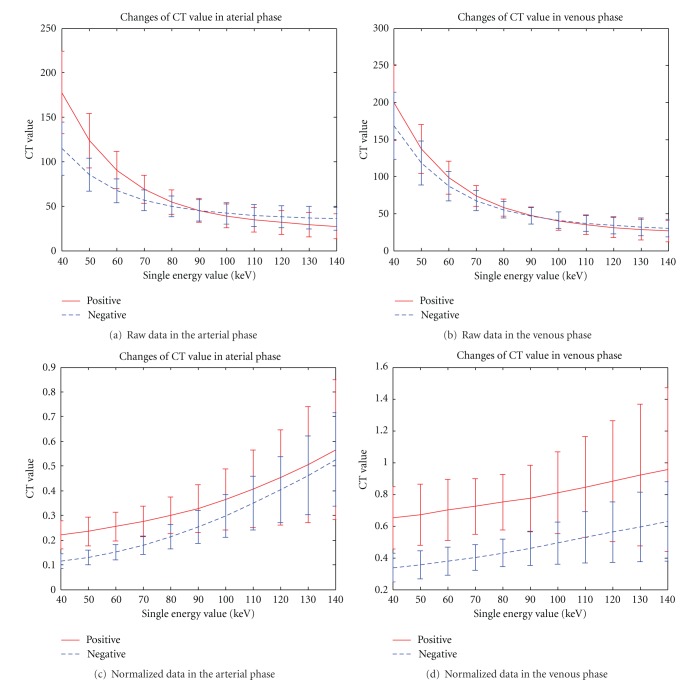
Monochromatic energy CT value in the arterial and venous phases of gastric lymph node metastasis.

Based on the statistical results of *r*_pb_, AUC, SU, and IG, compared with high monochromatic energy, low-energy features have higher relevance to lymph node metastasis according to clinical results. As shown in Figure 3, low-energy images display a large difference between lymph node metastasis (positive) and non-lymph node metastasis (negative), as monochromatic energy is associated with higher energies that yield less contrast between materials and more contrast with low energies. However, low-energy images bring more noise with higher contrast. Therefore, doctors usually select 70 keV as a tradeoff for clinical diagnosis.

#### 3.1.2. SFS-KNN Results

**Figure 4:**
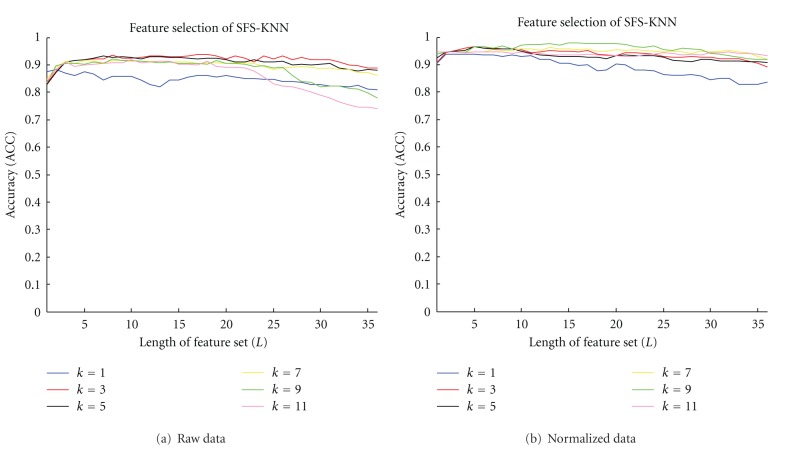
SFS-KNN feature selection procedures on raw and normalized data.

Figure 4 and Table 4 present the classification accuracy (ACC) of the KNN algorithm with different neighborhood sizes and the SFS algorithm with increasing lengths of the feature set. ACC first increases with the increasing length of the feature set, and then decreases. After application of the SFS algorithm, the feature set becomes shorter, whereas accuracy becomes higher compared with the original feature set that explains the effectiveness of SFS. From Table 4, we can examine ACC with different neighborhood sizes and selected features. When *K* = 5, the performance remains stable before and after data normalization, and ACC reaches 96.58% after normalization and finally selects 12 (effective-Z in the arterial phase), 30 (effective-Z in the venous phase), 31 (Calcium-Iodine in the venous phase), 33 (Iodine-Calcium in the venous phase), and 14 (Calcium-Water in the arterial phase) feature sets. These selected features are highly related to the classification results (lymph node metastasis). Among which the 12 (effective-Z in the arterial phase), 30 (effective-Z in the venous phase), 33 (Iodine-Calcium in the venous phase) feature sets are consistent with the pathology theory and clinical experience of doctors. As for the other feature sets, their effectiveness need to be further verified by studies. However, the SFS-KNN algorithm is not a global optimized solution, and it may lead to overfitting problems, which explain the decrease in ACC. In our experiments, the amount of the samples is not sufficient, so the large neighborhood size fails to reflect the local characteristics of the KNN classifier. Therefore, *K* = 9 is not selected as the optimal size.

#### 3.1.3. mRMR-KNN Results

**Figure 5:**
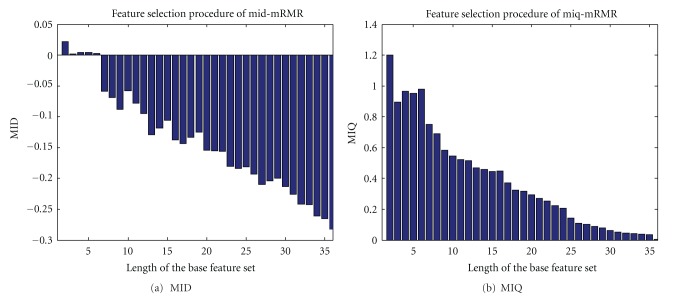
Feature selection procedures with different mRMR criteria.

Figure 5 shows two feature selection procedures with different mRMR criteria. Tables 5 and 6 reveal the classification performance of mRMR-KNN (MIQ and MID) with different neighborhood sizes [27]. We can see form the two tables that the two criteria of MIQ and MID acquire almost the same performances. After normalization, the accuracy with all different *K* are highly increased, thus demonstrate the positive effect of data normalization. Among the feature sets, we can conclude from the table that 15 (Iodine-Calcium in the arterial phase), 21 (60 keV in the venous phase), 30 (Effective-Z in the venous phase), and 3 (60 keV in arterial phase) are closely related to the lymph node metastasis, which highly agree with the pathology theory and clinical experience of doctors. With *K* = 5, the classification performance remains stable before and after normalization, which further verifies the optimal *K* (neighborhood size) value.

#### 3.1.4. Metric Learning Results

**Figure 6:**
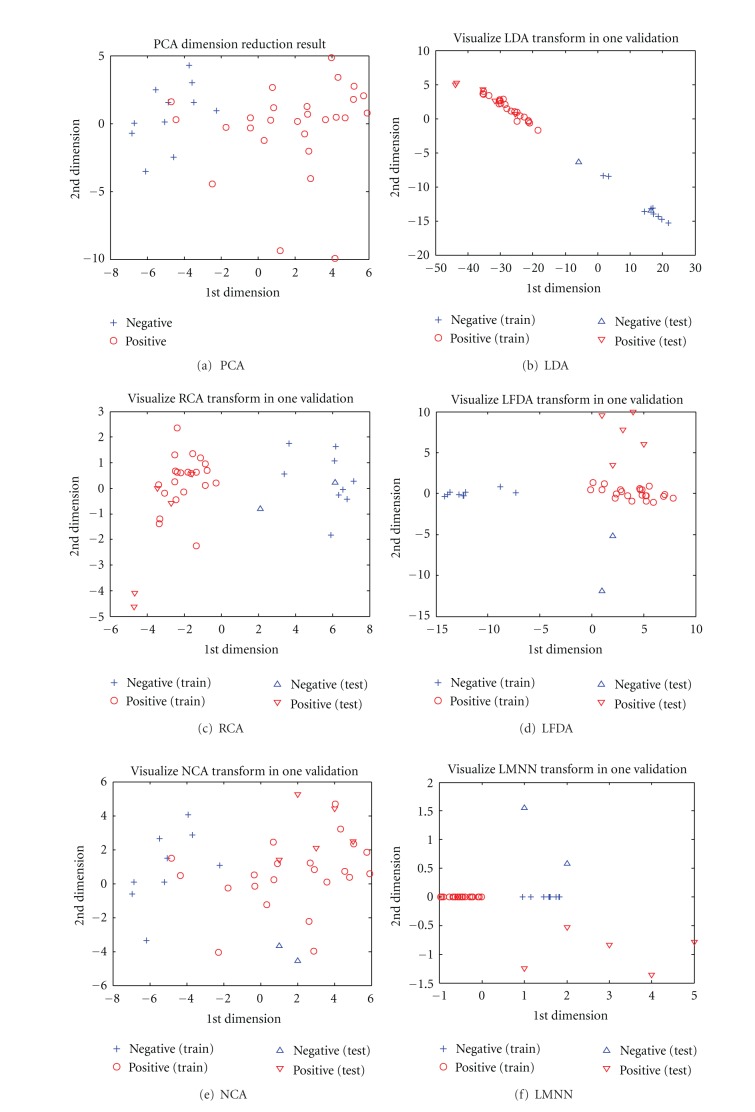
Dimension reduction results of different metric learning methods in one validation.

Figure 6 shows 2D visualized results of 6 different distance metric learning methods in one validation. In the two-dimensional projection space, the classes are better separated by the LDA transformation than by other distance metrics. However, the result of KNN with single distance metric is not very satisfying, that's why we consider combination.

Table 7 shows the classification accuracy of KNN algorithm with different-distance metric learning methods. Apparently, these results show that the data normalization helps a lot on classification. Moreover, PCA is a popular algorithm for data dimensionality reduction and operates in an unsupervised setting without using the class labels of the training data to derive informative linear projections. However, PCA can still have useful properties as linear preprocessing for KNN classification. By combining PCA with other supervised distance metric learning methods (e.g., LDA, RCA), we can obtain greatly improved performance. The accuracy of KNN classification depends significantly on the metric used to compute distances between different samples. 

### 3.2. Discussion

Based on the experimental results, the use of machine learning methods can improve the accuracy of clinical lymph node metastasis in gastric cancer. In our study, we mainly used the KNN algorithm for classification, which shows high efficiency. To improve effectiveness and classification accuracy, we first employed several feature-selection algorithms, such as mRMR and SFS methods, which both show an increase in accuracy. We obtained the highly related features of lymph node metastasis in accordance with the validated results of clinical pathology. Another way to improve accuracy is the use of distance metric learning for the input space of the data from a given collection of similar/dissimilar points that preserve the distance relation among the training data, and the application of the KNN algorithm in the new data patterns. Some schemes used in our experiments attained the overall accuracy of 96.33%.

## 4. Conclusions

The main contribution of our study is to prove the feasibility and the effectiveness of machine learning methods for computer-aided diagnosis (CAD) of lymph node metastasis in gastric cancer using clinical GSI data. In this paper, we employed a simple and classic algorithm called KNN that combines several feature selection algorithms and metric learning methods. The experimental results show that our scheme outperforms traditional diagnostic means (e.g., EUS and MDCT). 

One limitation of our research is the insufficient number of clinical cases. Thus, in our future work, we will conduct more experiments on clinical data to improve further the efficiency of the proposed scheme and to explore more useful and powerful machine learning methods for CAD in clinical. 

## Figures and Tables

**Table 1:** Distance metric learning methods used in this work.

Unsupervised distance metric learning method		Principal component analysis (PCA) [19, 21]
Supervised distance metric learning method	Global	Fisher discriminative analysis (FDA) [21]
		Relevant component analysis (RCA) [22]
	Local	Neighborhood component analysis (NCA) [23]
		Local fisher discriminative analysis (LFDA) [24]
		Large margin nearest neighborhood (LMNN) [25]

**Table 2:** Univariate analyses of the features of gastric lymph node metastasis arterial phase.

No.	Feature	Mean ± Standard		*P*1	*P*2	*r*_pb_	AUC	SU	IG
		Negative	Positive						
1	40 keV	114.97 ± 29.84	177.79 ± 46.25	0.000	0.000	0.569	0.875	0.174	0.186
2	50 keV	85.55 ± 18.81	123.69 ± 30.44	0.000	0.000	0.540	0.869	0.174	0.186
3	60 keV	67.49 ± 13.36	90.63 ± 21.15	0.000	0.002	0.488	0.845	0.186	0.208
4	70 keV	56.74 ± 11.53	68.93 ± 15.63	0.000	0.025	0.362	0.774	0.106	0.104
5	80 keV	49.93 ± 11.53	54.84 ± 13.68	0.001	0.302	0.172	0.596	0.070	0.071
6	90 keV	45.30 ± 12.05	45.27 ± 13.55	0.025	0.994	−0.001	0.502	0.001	0.001
7	100 keV	42.01 ± 12.18	39.08 ± 13.46	0.114	0.537	−0.103	0.552	0.013	0.015
8	110 keV	39.71 ± 12.37	34.68 ± 13.54	0.272	0.295	−0.174	0.599	0.014	0.015
9	120 keV	38.13 ± 12.56	31.62 ± 13.68	0.434	0.182	−0.221	0.623	0.025	0.027
10	130 keV	36.83 ± 12.73	29.25 ± 13.86	0.570	0.127	−0.252	0.653	0.079	0.086
11	140 keV	35.89 ± 12.86	27.41 ± 14.00	0.673	0.092	−0.277	0.660	0.079	0.086
12	Effective-Z	8.18 ± 0.26	8.71 ± 0.35	0.000	0.000	0.601	0.896	0.317	0.336
13	Calcium-Iodine	819.69 ± 10.39	810.02 ± 10.70	0.284	0.015	−0.391	0.754	0.126	0.127
14	Calcium-Water	14.05 ± 5.77	27.26 ± 9.12	0.000	0.000	0.594	0.899	0.315	0.343
15	Iodine-Calcium	−579.62 ± 8.65	−568.30 ± 9.31	0.000	0.001	0.500	0.822	0.174	0.165
16	Iodine-Water	10.10 ± 4.11	19.20 ± 6.22	0.000	0.000	0.596	0.896	0.315	0.343
17	Water-Calcium	1021.57 ± 15.74	1000.17 ± 18.21	0.000	0.002	−0.494	0.818	0.174	0.165
18	Water-Iodine	1030.55 ± 13.74	1017.24 ± 15.20	0.291	0.017	−0.386	0.734	0.174	0.165

**Table 3:** Univariate analyses of the features of gastric lymph node metastasis venous phase.

No.	Feature	Mean ± Standard		*P*1	*P*2	*r*_pb_	AUC	SU	IG
		Negative	Positive						
19	40 keV	168.56 ± 45.67	199.95 ± 51.33	0.000	0.087	0.282	0.684	0.070	0.072
20	50 keV	117.94 ± 29.61	137.13 ± 32.85	0.000	0.102	0.269	0.673	0.070	0.072
21	60 keV	86.91 ± 20.03	98.71 ± 22.14	0.000	0.135	0.247	0.653	0.086	0.092
22	70 keV	67.54 ± 13.52	73.94 ± 14.14	0.000	0.209	0.209	0.620	0.106	0.104
23	80 keV	55.09 ± 11.10	57.96 ± 11.31	0.000	0.481	0.118	0.559	0.110	0.122
24	90 keV	46.76 ± 10.95	47.02 ± 11.50	0.000	0.949	0.011	0.535	0.018	0.018
25	100 keV	41.08 ± 11.04	39.90 ± 12.06	0.000	0.781	−0.047	0.562	0.018	0.020
26	110 keV	37.08 ± 11.29	34.81 ± 12.73	0.003	0.611	−0.085	0.599	0.011	0.011
27	120 keV	34.25 ± 11.56	31.27 ± 13.30	0.012	0.521	−0.107	0.613	0.011	0.011
28	130 keV	32.10 ± 11.86	28.56 ± 13.79	0.028	0.461	−0.123	0.613	0.011	0.011
29	140 keV	30.37 ± 12.09	26.42 ± 14.19	0.052	0.423	−0.134	0.626	0.018	0.019
30	Effective-Z	8.61 ± 0.38	8.87 ± 0.42	0.000	0.081	0.286	0.680	0.087	0.088
31	Calcium-Iodine	812.48 ± 9.36	807.83 ± 11.88	0.651	0.254	−0.190	0.643	0.025	0.026
32	Calcium-Water	24.65 ± 9.57	31.44 ± 11.52	0.000	0.093	0.276	0.673	0.086	0.087
33	Iodine-Calcium	−570.78 ± 8.89	−565.23 ± 11.43	0.005	0.159	0.233	0.650	0.037	0.042
34	Iodine-Water	17.56 ± 6.43	22.25 ± 7.71	0.000	0.084	0.284	0.667	0.074	0.073
35	Water-Calcium	1005.60 ± 17.73	994.85 ± 22.22	0.011	0.162	−0.231	0.657	0.037	0.042
36	Water-Iodine	1021.14 ± 13.95	1014.62 ± 17.03	0.631	0.270	−0.184	0.636	0.011	0.011

**Table 4:** Classification performance of the SFS-KNN algorithm with different neighborhood sizes.

Neighborhood size		*K* = 1	*K* = 3	*K* = 5	*K* = 7	*K* = 9
Pre-norm	Selected features	14, 16	14, 31, 5, 15, 26, 4, 27, 21, 24, 9, 32, 2, 25, 8, 28, 3, 16	14, 31, 10, 36, 3, 25, 2	12, 31, 8, 29, 3, 15, 33, 1	12, 31, 23, 26, 3, 24, 30, 16
	Accuracy	88.29%	93.68%	93.29%	91.71%	92.24%

Norm	Selected features	12, 30	20, 15, 11, 30, 5	12, 30, 31, 33, 14	12, 19, 20, 30, 5, 18, 25, 17, 34, 3, 32, 15, 24	12, 19, 29, 30, 8, 34, 33, 25, 15, 6, 24, 7, 10, 20, 17
	Accuracy	93.95%	96.45%	96.58%	96.18%	97.89%

**Table 5:** Classification performance of mRMR-KNN (MIQ) with different neighborhood sizes.

	Neighorhood size	*K* = 1	*K* = 3	*K* = 5	*K* = 7	*K* = 9
Prenorm	Sequence	14, 19, 5, 17, 23, 12, 3, 16, 18, 22, 1, 15, 4, 2, 30, 13, 21, 32, 10, 33, 11, 34, 20, 35, 31, 25, 9, 29, 24, 8, 7, 26, 36, 27, 28, 6				
	Length	1	28	28	35	1
	Accuracy	87.50%	89.74%	89.08%	87.24%	81.71%
Norm	Sequence	15, 21, 3, 30, 17, 24, 12, 14, 23, 5, 16, 22, 2, 18, 27, 1, 20, 4, 33, 25, 13, 19, 6, 28, 35, 26, 32, 7, 29, 34, 8, 31, 9, 11, 10, 36				
	Length	4	2	2	2	10
	Accuracy	90.00%	94.87%	94.87%	94.74%	95.66%

**Table 6:** Classification performance of mRMR-KNN (MID) with different neighborhood sizes.

	Neighborhood size	*K* = 1	*K* = 3	*K* = 5	*K* = 7	*K* = 9
Prenorm	Sequence	12, 26, 22, 18, 3, 30, 14, 6, 19, 16, 36, 2, 17, 5, 1, 24, 35, 15, 23, 4, 34, 13, 29, 21, 7, 31, 11, 32, 25, 20, 9, 28, 33, 10, 8, 27				
	Length	1	26	26	26	20
	Accuracy	87.50%	90.39%	89.34%	87.11%	82.50%
Norm	Sequence	15, 21, 2, 30, 24, 17, 5, 14, 23, 12, 18, 22, 27, 4, 16, 33, 7, 1, 20, 25, 13, 3, 29, 19, 6, 35, 28, 31, 8, 32, 26, 11, 34, 36, 9, 10				
	Norm	Length	4	2	2	16
	Accuracy	89.74%	94.87%	94.87%	95.66%	95.26%

**Table 7:** Classification performance of the KNN algorithm with metric learning methods.

Data (length of feature set)	Prenorm (4)	Norm 01 (5)
KNN	80.79%	83.68%
without PCA	80.79%	83.68%
PCA	82.11%	81.84%
PCA + LDA	77.89%	96.33%
PCA + RCA	77.63%	96.33%
PCA + LFDA	76.97%	96.33%
PCA + NCA	76.58%	86.32%
PCA + LMNN	76.84%	96.33%
